# Antitumor effect of YM155, a novel small-molecule survivin suppressant, via mitochondrial apoptosis in human MFH/UPS

**DOI:** 10.3892/ijo.2015.3077

**Published:** 2015-07-09

**Authors:** MASAYA MINODA, TERUYA KAWAMOTO, TAKESHI UEHA, ETSUKO KAMATA, MASAYUKI MORISHITA, RISA HARADA, MITSUNORI TODA, YASUO ONISHI, HITOMI HARA, MASAHIRO KUROSAKA, TOSHIHIRO AKISUE

**Affiliations:** 1 Department of Orthopaedic Surgery, Kobe University Graduate School of Medicine, Chuo-ku, Kobe 650-0017; 2 Division of Rehabilitation Medicine, Kobe University Graduate School of Medicine, Chuo-ku, Kobe 650-0017; 3 Department of Rehabilitation Science, Kobe University Graduate School of Health Sciences, Suma-ku, Kobe 654-0142, Japan

**Keywords:** survivin, apoptosis, YM155, malignant fibrous histiocytoma/undifferentiated pleomorphic sarcoma, sarcoma

## Abstract

Survivin is a member of the inhibitor of apoptosis family, which is known to inhibit mitochondrial apoptosis. Survivin is highly expressed in cancers and plays an important role in cancer cell survival, and increased survivin expression is an unfavorable prognostic marker in cancer patients. YM155, a novel small-molecule survivin suppressant, selectively suppresses survivin expression, resulting in the induction of apoptosis in various malignancies. However, the roles of survivin in human malignant fibrous histiocytoma/undifferentiated pleomorphic sarcoma (MFH/UPS) have not been studied. In the present study, we examined survivin expression in human musculoskeletal tumor tissues, and the effect of survivin inhibition by siRNA or YM155 on apoptotic activity in human MFH/UPS cell lines. In tumor tissues, mRNA expression of survivin was significantly higher in MFH/UPS samples than in benign schwannomas. Moreover, *in vitro* studies revealed that both survivin siRNA and YM155 suppressed survivin expression and inhibited MFH/UPS cell proliferation in a dose- and a time-dependent manner. Further, the numbers of apoptotic cells significantly increased with YM155 treatment. *In vivo*, tumor volume in YM155-treated groups was significantly reduced without significant bodyweight loss. Increased apoptotic activity along with decreased survivin expression was also observed in YM155-treated tumors. The findings in this study strongly suggest that survivin suppressants, including YM155, contribute to the suppression of human MFH/UPS cell growth via promoting mitochondrial apoptosis, and that survivin may be a potent therapeutic target for the novel treatment of human MFH/UPS.

## Introduction

Musculoskeletal malignancies, particularly high-grade sarcomas such as malignant fibrous histiocytoma (MFH), which has recently been classified as undifferentiated pleomorphic sarcoma (UPS), are clinically aggressive and demonstrate high metastatic behavior (1,2). Recent advances have led to multidisciplinary treatments for musculoskeletal malignancies, including surgery, chemotherapy, and radiation therapy, resulting in great improvement in the quality of life of affected patients. Although many chemotherapeutic protocols are used to treat human sarcomas, current therapeutic strategies for high-grade sarcomas, except Ewing's sarcomas and the peripheral primitive neuroectodermal tumors (PNET) family, are ineffective, and result in poor patient prognosis due to local recurrence and metastases (3). Therefore, there is a great need to understand the mechanisms of tumor progression, and new therapeutic strategies against high-grade sarcomas are required.

Survivin is a member of the inhibitor of apoptosis (IAP) family, which is known to inhibit mitochondrial apoptosis. It is expressed in a wide range of embryonic and fetal tissues, but is undetectable in terminally differentiated normal adult tissues, and is highly expressed in most solid and hematological malignancies (4–6). Survivin has been implicated in both cell survival and the regulation of mitosis in tumor cells, and has been consistently identified by molecular profiling analyses to be associated with more advanced disease, abbreviated survival, accelerated rates of recurrence, and resistance to chemotherapy and radiation therapy (7–10). Moreover, elevated survivin expression is an unfavorable prognostic marker correlating with decreased overall survival in a variety of malignant tumors (11). However, there have been few studies that examine survivin expression in musculoskeletal malignancies (12–16).

YM155 is a novel small-molecule that selectively suppresses survivin gene expression, resulting in activation of caspases and induction of apoptosis in malignant tumors (17). The molecular mechanisms underlying YM155-mediated survivin suppression are under evaluation through identification of YM155-interacting molecules that bind to promoter regions of the survivin gene (4). Recently, clinical studies have investigated the effects of using YM155 to selectively target survivin on certain cancers (18–20). However, studies have not focused on the effects of survivin suppressants on musculoskeletal malignancies like MFH/UPS.

Based on previous studies, we hypothesized that survivin is highly expressed in human MFH/UPS, and that survivin contributes to tumor progression by inhibiting mitochondrial apoptosis in human MFH/UPS. In the present study, we examined survivin expression in human MFH/UPS tumor tissues and evaluated the effect of survivin inhibition on cell apoptosis using human MFH/UPS cell lines *in vitro*. For *in vivo* studies, we used YM155 to characterize the preclinical efficacy profile of survivin.

## Materials and methods

### Musculoskeletal tumor tissue samples and human MFH/UPS cell lines

We used 30 human musculoskeletal tumor tissue samples including 15 benign schwannomas (as control) and 15 MFH/UPS samples. This study was approved by the Kobe University Hospital ethics committee (permission no. 1077), and all patients provided informed consent prior to surgery. The samples were obtained by surgery at Kobe University Hospital in accordance with institutional guidelines and immediately stored at −80°C until use.

Three human MFH/UPS cell lines (Nara-H, Nara-F and TNMY1) were studied *in vitro* and *in vivo*. Nara-H and Nara-F were obtained from ScienStuff Co. (Nara, Japan) (21), and TNMY1 was previously established in our laboratory (22). Cells were grown in Dulbecco's modified Eagle's medium (DMEM; Sigma-Aldrich Co., St. Louis, MO, USA) supplemented with 10% (v/v) fetal bovine serum (Sigma-Aldrich) and 100 U/ml penicillin/streptomycin solution (Sigma-Aldrich). Cell lines were routinely maintained at 37°C in a humidified 5% CO_2_ atmosphere. For all experiments, we used DMEM containing 10% FBS without the antibiotic solution.

### siRNA knockdown of survivin in human MFH/UPS cells

To assess the effects of survivin inhibition on apoptotic activity and cell proliferation in human MFH/UPS cells *in vitro*, we performed siRNA transfection with either survivin-siRNA (Ambion Inc., Austin, TX, USA) or non-specific control siRNA (Ambion Inc.) in MFH/UPS cell lines using Lipofectamine™ 2000 Transfection reagent, according to the manufacturer's instructions (Invitrogen, Carlsbad, CA, USA). Efficacy of survivin knockdown was assessed by quantitative real-time PCR (qPCR).

### Survivin suppressant, YM155

YM155 was purchased from AdooQ BioScience (Irvine, CA, USA), dissolved in dimethyl sulfoxide, and immediately stored at −80°C. This stock solution was diluted into culture medium and saline for *in vitro* and *in vivo* experiments, respectively, immediately before use.

### Human MFH/UPS xenograft studies

Male BALB/c nude mice, aged 5 weeks, were obtained from CLEA Japan, Inc. (Tokyo, Japan). The animals were maintained under pathogen-free conditions, in accordance with institutional principles. All animal experiments were approved by Kobe University Animal Experimentation Regulations (permission no. P-130807). Nara-H cells were implanted into the dorsal, subcutaneous area of mice (n=18) at a dose of 1.0×10^6^ cells in 500 μl PBS, as previously described (23) and mice were randomly divided into three groups: YM155 at 4 mg (n=6), YM155 at 2 mg (n=6) and control (n=6). Treatment commenced 2 weeks after cell implantation by intraperitoneal injection of YM155 (2 or 4 mg/kg) or saline (as control), five times/week for 2 weeks. Tumor volume was calculated, as previously described, according to the formula V = π/6 × a^2^ × b, where a and b represent the shorter and the longer dimensions of the tumor, respectively (23). At the end of the experiments, all tumors were excised and stored at −80°C. Survivin expression was assessed by qPCR, and apoptotic activity was evaluated by FACS and immunofluorescence staining.

### Quantitative real-time PCR (qPCR)

We isolated total RNAs from tumor tissues, cells or implanted tumors using an RNeasy Mini kit, according to the manufacturer's protocol (Qiagen, Valencia, CA, USA), and first-strand cDNAs were synthesized using a High Capacity cDNA Transcription kit (Applied Biosystems, Foster City, CA, USA). qPCR was performed in a 20-μl reaction mixture using the Power SYBR Green Master Mix reagent (Applied Biosystems) on an ABI PRISM 7500 sequence detection system (Applied Biosystems). The cycling conditions were as follows: 1 cycle at 95°C for 10 min followed by 40 cycles at 95°C for 15 sec and 60°C for 1 min. Primers for human survivin [5′-CTT GGC CCA GTG TTT CTT CT-3′ (upstream) and 5′-CCT CCC AAA GTG CTG GTA TT-3′ (downstream)] and the internal control, human β-actin [5′-AGT CCT GTG GCA TCC ACG AAA-3′ (upstream) and 5′-GTC ATA CTC CTG CTT GCT GA-3′ (downstream)] were synthesized by Applied Biosystems. The values were normalized with β-actin, and relative expression was analyzed using the ΔΔCt method.

### Immunoblot analysis

Lysates were extracted from cells or implanted tumors using a whole cell lysis buffer (Mammalian Protein Extraction reagent, Thermo Scientific, Rockford, IL, USA) supplemented with a protease and phosphatase inhibitor mix (Roche Applied Science, Indianapolis, IN, USA). Protein content was quantified using the BCA Protein Assay reagent (Bio-Rad, Hercules, CA, USA). Samples containing equal amounts of protein were electrophoresed through 7.5–15% SDS-PAGE gradient gels and transferred onto PVDF membranes. After blocking, membranes were incubated overnight at 4°C with the following antibodies in CanGet Signal Solution 1 (Toyobo Co., Ltd., Osaka, Japan): anti-human survivin (1:1,000), anti-human PARP (1:1,000), anti-human cleaved PARP (1:1,000), anti-human caspase-3 (1:1,000), anti-human cleaved caspase-3 (1:500), anti-human caspase-9 (1:1,000), and anti-human cleaved caspase-9 (1:500). All antibodies were purchased from Cell Signaling Technology (Denvers, MA, USA). Following washes, membranes were incubated with the appropriate secondary antibody conjugated to horseradish peroxidase and were exposed with ECL Plus western blot detection system reagent (GE Healthcare Biosciences, Piscataway, NJ, USA). Protein expression was detected by Chemilumino analyzer LAS-3000 mini (Fujifilm, Tokyo, Japan). Membranes were reprobed with anti-human α-tubulin antibody (Sigma-Aldrich) to confirm equal protein loading.

### DNA fragmentation assays

DNA fragmentation was assessed by FACS analysis using the APO-DIRECT kit, according to the manufacturer's protocol (BD Pharmingen, Franklin Lakes, NJ, USA) (9). To obtain a single cell suspension from implanted tumor tissues, tumors were excised, minced, and filtered through a cell strainer (BD Falcon, Bedford, MA, USA). Erythrocytes were lysed in BD Pharm Lyse™ Lysing Buffer (BD Pharmingen) and the remaining cells were pelleted and resuspended in PBS. Single cell suspensions were fixed in 1% (v/v) paraformaldehyde and resuspended in 70% (v/v) ice cold ethanol at a concentration of 1.0×10^6^ cells/ml. Each cell pellet was resuspended in 50 μl of DNA labeling solution (reaction buffer, 10 μl; TdT enzyme, 0.75 μl; FITC-dUTP, 8.0 μl; dH_2_O, 31.25 μl) and incubated for 60 min at 37°C. FITC-dUTP-labeled cells were analyzed.

### Cell proliferation assays

We assessed cell proliferative activities in siRNA-transfected or YM155-treated cells by WST-8 assay using Cell Counting Kit-8 (CCK-8; Dojindo Inc., Kumamoto, Japan). Cells were seeded in 96-well culture plates at a density of 5×10^3^ cells/well in 100 μl culture medium. At the indicated times after siRNA transfection or YM155, 10 μl of the CCK-8 solution was added, and optical density was measured at 450 nm using a Model 680 Microplate Reader (Bio-Rad). The relative number of viable cells in each well was calculated.

### Immunofluorescence staining

Formalin-fixed, paraffin-embedded tumor sections (5 mm) were pretreated with citrate buffer for 40 min at 95°C, quenched with 0.05% H_2_O_2_, and incubated with primary antibody overnight at 4°C. Apoptotic activity was assessed in treated tumors by immunofluorescence staining using the APO-DIRECT kit, following the manufacturer's protocol (BD Pharmingen). The nuclei were stained with DAPI. The images were obtained using a BZ-8100 confocal microscope (Keyence, Osaka, Japan).

### Statistical analysis

Each experiment was performed independently at least three times. ANOVA with post hoc test was used to compare for continuous values. All tests were considered significant at p<0.05. Data were presented as the mean ± SE. For distributed data, the two-tailed Mann-Whitney U test was used for comparison between groups.

## Results

### Survivin expression is higher in MFH/UPS tissues

**Figure 1:**
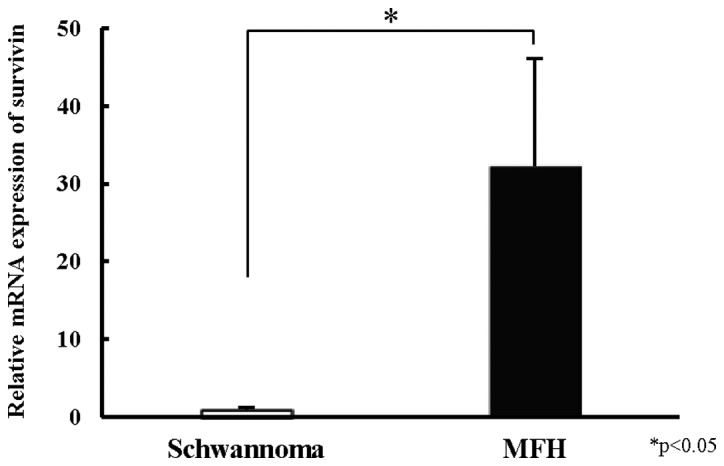
Evaluation of survivin mRNA expression in human musculoskeletal tumor tissue samples. The relative survivin mRNA expression was evaluated in 15 benign schwannomas and 15 MFH/UPSs by qPCR. Results were normalized to the mean value of the schwannomas. Data represent mean ± SE of at least three independent experiments (^*^p<0.05).

We first surveyed survivin mRNA expression in human muscloskeletal tumor tissue samples, and found that survivin mRNA expression in MFH/UPS samples was significantly higher than expression in samples from benign schwannomas. The relative expression of survivin increased 32-fold in MFH/UPS samples compared to that in benign schwannomas (^*^p<0.05, Fig. 1).

### siRNA knockdown of survivin affects mitochondrial apoptosis and cell proliferation

**Figure 2:**
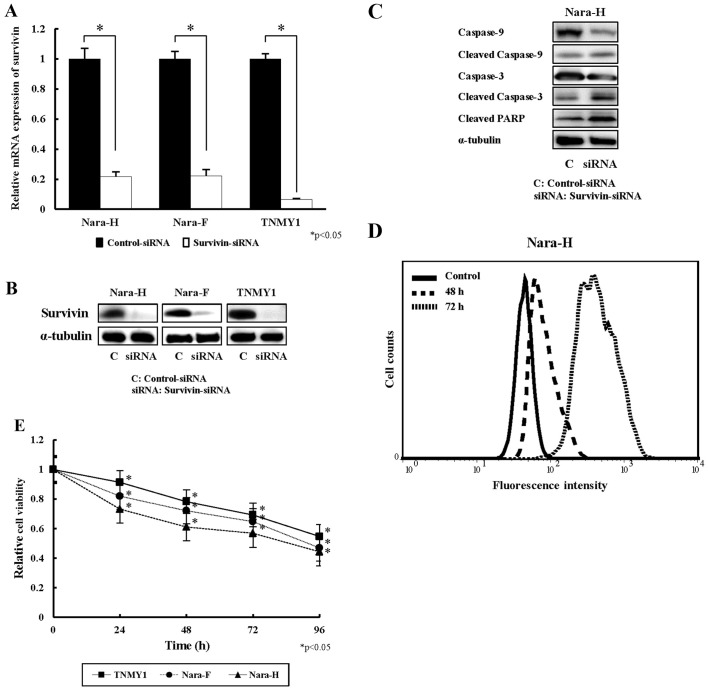
Effects of survivin siRNA on apoptotic activity and cell viability in human MFH/UPS cell lines. (A) Survivin mRNA expression was evaluated by qPCR in siRNA transfected MFH/UPS cell lines after 72 h of siRNA transfection. Data represent mean ± SE of at least three independent experiments (^*^p<0.05). (B) Immunoblot analysis for survivin in siRNA-transfected MFH/UPS cell lines. (C) Immunoblot analysis for apoptosis-related proteins in siRNA-transfected Nara-H MFH/UPS cells. (D) DNA fragmentation was assessed by FACS analysis in siRNA-transfected Nara-H MFH/UPS cells. (E) Relative cell viability was assessed by WST-8 assay in siRNA-transfected MFH/UPS cell lines after 24, 48, 72, and 96 h of siRNA transfection. Data represent mean ± SE of at least three independent experiments (^*^p<0.05).

In qPCR, survivin-siRNA transfection significantly suppressed survivin mRNA expression in human MFH/UPS cells compared to control siRNA (^*^p<0.05, Fig. 2A). Consistent with these results, immunoblot analyses showed that survivin protein expression decreased with survivin-siRNA transfection (Fig. 2B).

To evaluate the effects of survivin-siRNA on cellular apoptosis, we assessed DNA fragmentation and the expression of apoptosis-related proteins in siRNA-transfected Nara-H cells. Immunoblot analyses indicated that the expression of the cleaved forms of caspase-3, -9, and PARP increased in survivin-siRNA transfected cells 72 h post-transfection, while the expression of those proteins in control cells was weakly detected (Fig. 2C). In FACS analysis, DNA fragmentation increased in survivin-siRNA transfected cells in a time-dependent manner when compared to control cells (Fig. 2D). To identify the effect of survivin knockdown on MFH/UPS cell viability, we performed cell proliferation assays in siRNA-transfected cells. In all three MFH/UPS cell lines, cell viabilities were significantly decreased in a time-dependent manner (^*^p<0.05, Fig. 2E).

### YM155 increases apoptotic activity in human MFH/UPS cells in vitro

**Figure 3:**
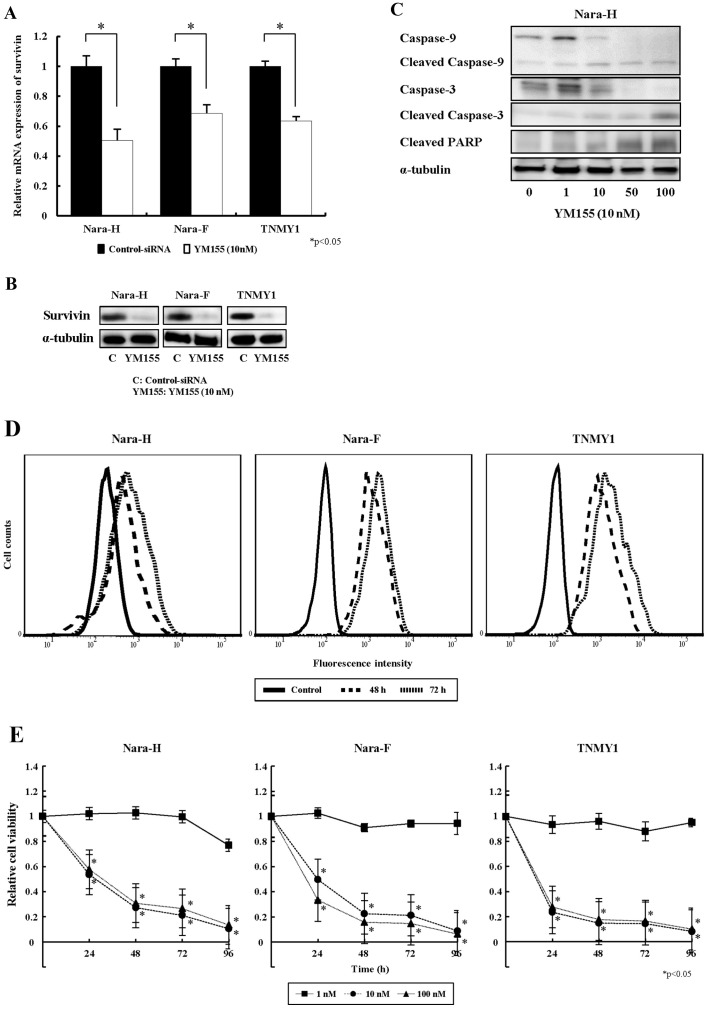
Effects of YM155 on apoptotic activity and cell viability in human MFH/UPS cell lines. (A) Survivin mRNA expression was evaluated by qPCR after 72 h of YM155 treatment (10 nM). Data represent mean ± SE of at least three independent experiments (^*^p<0.05). (B) Protein expression of survivin was assessed by immunoblot analysis in 10 nM of YM155-treated human MFH/UPS cell lines. (C) Immunoblot analysis for apoptosis-related proteins in YM155-treated Nara-H cells. (D) DNA fragmentation after 48 and 72 h of YM155 treatment was assessed by FACS analysis in human MFH/UPS cell lines. (E) Relative cell viability was assessed by WST-8 assay in YM155 treated MFH/UPS cell lines after 24, 48, 72, and 96 h of treatment. Data represent mean ± SE of at least three independent experiments (^*^p<0.05).

YM155 (10 nM) strongly suppressed mRNA (^*^p<0.05, Fig. 3A) and protein (Fig. 3B) expression of survivin in human MFH/UPS cell lines after 72 h of treatment. In immunoblot analyses, expression of the cleaved forms of caspase-3, -9, and PARP increased in YM155-treated Nara-H cells in a dose-dependent manner, while expression of the full length forms of caspase-3 and caspase-9 decreased (Fig. 3C). Consistent with this, DNA fragmentation increased in all YM155-treated MFH/UPS cell lines in a time-dependent manner (Fig. 3D). Additionally, cell proliferation assays showed that YM155, at concentrations of 10 nM or more, significantly decreased cell proliferation in a dose-and a time-dependent manner in all three MFH/UPS cell lines (Fig. 3E).

### Antitumor effects of YM155 on human MFH/UPS xenografts in vivo

**Figure 4:**
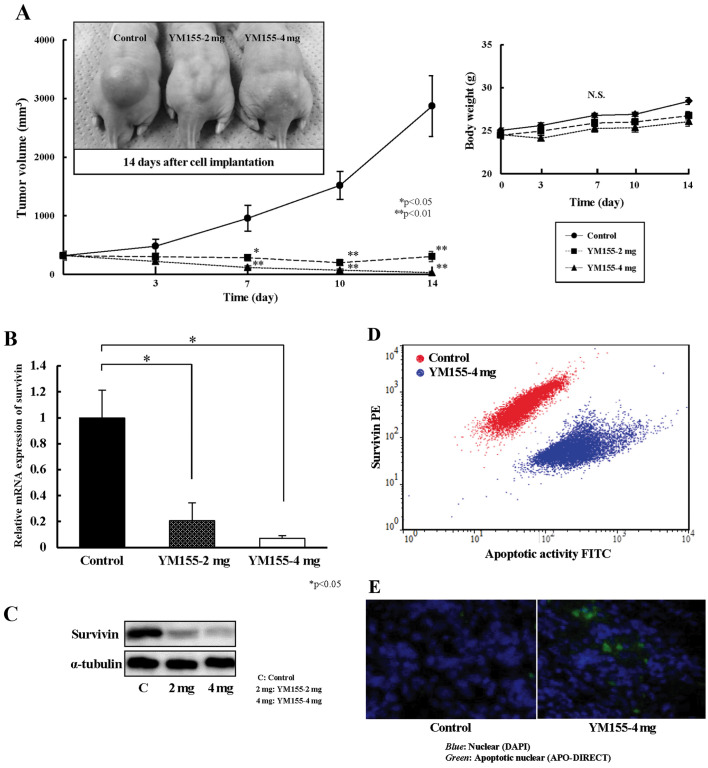
*In vivo* antitumor activities of YM155 on human MFH/UPS xenografts (Nara-H). (A) Tumor volume (mm^3^) and body weight (g) in YM155-treated (2 mg or 4 mg) or control mice was monitored for 14 days. Data represent mean ± SE of at least three independent experiments (^*^p<0.05, ^**^p<0.01). (B) Survivin mRNA expression in YM155-treated or control tumors was evaluated by qPCR. Data represent mean ± SE of at least three independent experiments (^*^p<0.05). (C) Immunoblot analysis for survivin in YM155-treated or control tumors. (D) Correlation between apoptotic activity and survivin expression in YM155-4 mg-treated or control tumors was assessed by FACS analysis (red, control; blue, YM155-4 mg). (E) DNA fragmentation analysis of tumor tissues from YM155-4 mg-treated and control mice by immunofluorescence staining [blue, nuclear (DAPI); green, apoptosis nuclear (APO-DIRECT)].

We evaluated the *in vivo* antitumor activity of YM155 using human MFH/UPS xenografts. The final tumor volumes in the YM155 at 2 mg-treated and YM155 at 4 mg-treated groups were 10.7 and 1.1%, respectively, of that in the control group (p<0.05). No significant loss in body weight was observed during the experimental periods (Fig. 4A). Survivin mRNA (p<0.05, Fig. 4B) and protein (Fig. 4C) expression in YM155 treated tumors significantly decreased compared with control tumors. Using FACS analysis (Fig. 4D) and immunofluorescence staining (Fig. 4E), we found that apoptotic activity significantly increased after YM155 treatment, while survivin expression decreased.

## Discussion

MFH/UPSs are clinically aggressive and highly metastatic (1,2). Current therapeutic strategies for treating soft tissue sarcomas (STS) are ineffective, and patient prognosis is poor due to local recurrence and distant metastases (3). STSs are histologically a broad group of rare tumors, accounting for 1% of all human malignant tumors. Radical surgery remains the primary mode of treatment for STSs; however, adjuvant chemotherapy or radiation therapy are desired early stage alternatives due to the high recurrence and metastatic rate of the tumor. Among the approved antitumor drugs, doxorubicin- and ifosfamide-based cytotoxic chemotherapies play an established role for STSs. Moreover, identification of the molecular pathways involved in STS tumorigenesis led to the development of molecular targeting agents which mainly inhibit tyrosine kinases, such as imatinib, used for advanced or metastatic dermatofibrosarcoma protuberans; pazopanib, approved as a second line regimen for advanced non-adipocitic STSs; and sunitinib, used for solitary fibrous tumor, alveolar soft part sarcoma, and extraskeletal myxoid chondrosarcoma (24,25). Chemotherapeutic effects should be important in the survival of patients with the disease, and this systemic modality should be the treatment of choice for patients at an advanced stage (26); however, the conventional chemotherapeutic strategies for high-grade STSs are ineffective and the prognosis of patients can be extremely poor because of local recurrence and metastases (3). Although studies have shown beneficial effects for tyrosine kinase inhibitors, new therapeutic strategies focusing on other molecular targets are required to be effective against high-grade STSs.

In this study, we examined the therapeutic potential of the inhibition of survivin, a member of the IAP family, on human MFH/UPS tumor growth. Survivin, the smallest member of the IAP family, is a 142-amino acid, 16.5-kDa protein coded by a single-copy gene on the human 17q25 chromosome. Structurally, survivin contains a single repeat of the characteristic baculovirus IAP domain, essential for the caspase-inhibitory function, and an extended carboxy-terminal α-helical coiled coil, but does not contain other identifiable domains (27–29). Resistance to apoptosis is a hallmark of malignant tumors; overexpression of IAP proteins enhances resistance to apoptotic stimuli in various malignancies (30). Among this protein family, survivin has taken a center stage because of its markedly specific expression in cancer cells (8,28). In the present study, we surveyed survivin expression in human musculoskeletal tumor tissue samples, and found that survivin was highly expressed in human MFH/UPS. This result suggests that elevated survivin expression strongly influences tumor progression in malignant musculoskeletal tumors.

Survivin is a bi-functional IAP that has been implicated in protection from apoptosis and regulation of mitosis (31). Initially, survivin was described as an inhibitor of caspase-9 (32); however, several studies have shown that the role of survivin in cancer pathogenesis is not limited to the inhibition of apoptosis but also involves regulation of the mitotic spindle checkpoint, and promotion of angiogenesis and chemoresistance (28,33–36). Although many functions for survivin have been identified, the mechanism by which survivin inhibits apoptosis has remained elusive. In particular, it is not clear whether survivin has a genuine function in the inhibition of apoptosis that is independent of its role as a master regulator of mitosis, and whether this function is important in disease pathogenesis. Recent experiments show that survivin inhibits active caspase-9 but not active caspase-3 and -7, and that this inhibition requires the IAP family member, X-linked IAP (XIAP) (35), and the hepatitis B X-interacting protein (37).

While undetectable in most adult differentiated tissues, survivin is ubiquitously expressed during embryonic development and is highly re-expressed in malignant tumors, including lung, breast, colorectal, gastric, prostate, hepatocellular, and renal cancers, as well as melanoma and STS (13,15,38–40). Studies suggest an interrelation between survivin expression and either poor disease prognosis or unresponsiveness to chemotherapy or radiation therapy (39,41,42). These findings strongly suggest that suppressing survivin may substantially contribute to antitumor activity in cancer cells. Tsuji *et al* reported that dsRNA-mediated silencing of survivin noticeably reduces survivin mRNA and protein expression in the pancreatic cancer cell line PANC-1, thereby resulting in cell apoptosis while failing to influence the cell cycle (43). Wang *et al* showed that conjugate-mediated survivin-siRNA can efficiently target glioma tumors *in vitro* and *in vivo*, and that silencing survivin by this method prolonged the survival times of orthotopic tumor-bearing mice (44). In musculoskeletal malignancies, the survivin inhibition by survivin-specific siRNAs enhanced sensitivity to doxorubicin and cisplatin in a survivin-overexpressing osteosarcoma cell line (45). In the present study, we found that survivin-siRNA strongly suppressed survivin expression, and that survivin-siRNA significantly increased the number of apoptotic cells and decreased cell proliferation in human MFH/UPS cell lines. Therefore, survivin inhibition likely induces cell apoptosis, resulting in an antitumor effect on human MFH/UPS.

YM155, a novel small-molecule suppressor of survivin, suppresses survivin expression with little effect on the expression levels of other IAP family members. The exact molecular mechanism by which YM155 downregulates survivin expression at the mRNA level and, subsequently, at the protein level, is not known. A previous study indicated that YM155 treatment inhibits the growth of wide variety of cancer cell lines *in vitro* and demonstrates significant antitumor activity without causing body weight loss in xenograft models (4). The study indicated that tumor regression induced by YM155 was associated with reduced intratumoral survivin expression levels, increased apoptosis, and decreased mitotic index (4). In the present study, we demonstrated that YM155 treatment induced mitochondrial apoptosis and decreased cell proliferation in human MFH/UPS cells *in vitro*, and that YM155 suppressed *in vivo* MFH/UPS cell growth by inducing apoptosis without apparent body weight loss. Our results strongly suggest that survivin expression contributes to human MFH/UPS tumor progression, and that survivin inhibition by either specific siRNA or by YM155, a novel survivin suppressant, provides an antitumor effect on human MFH/UPS via induction of apoptosis.

Considering that apoptosis is the primary mode of cell death induced by several classes of anticancer agents, a possible general role of survivin in determining the chemo-sensitivity profiles of tumor cells has been investigated and concluded that the suppression of survivin expression achieved increased sensitivity of various anticancer agents (39,46,47). Faversani *et al* reported that high-throughput pharmacologic targeting of survivin family proteins with YM155 selectively potentiated the effect of doxorubicin, and induced tumor cell apoptosis in breast cancer cell types. This result suggests that incorporation of YM155 in anthracycline-containing chemotherapy may result in greater clinical activity across breast cancer subtypes, and potentially overcome constitutive treatment resistance (48). Clinically, there are several ongoing studies using YM155 (18,19). Tolcher *et al* reported in a phase II study of YM155 that 25% of patients with castration-resistant prostate cancer had prolonged stable disease and that the tolerability regimen induced responses in the phase I trial. Survivin-targeting therapies can potentially activate the apoptotic pathways and induce mitochondrial activation (49). The first phase I/II study of YM155 with paclitaxel and carboplatin in patients with advanced non-small cell lung cancer (NSCLC) indicated that the administration of YM155 in combination with carboplatin and paclitaxel every 3 weeks resulted in a favorable safety profile but did not appear to significantly improve the response rate in advanced NSCLC. The authors concluded that combinations targeting several components within apoptotic pathways may be more effective (18). In the present study, we have proved that survivin inhibition shows antitumor effect on human MFH/UPS cells. Therefore, further *in vivo* studies should be conducted to test the efficacy of YM155 in the treatment of STS when used in combination therapy and we have no doubt that we can get better results particularly by the combination with doxorubicin.

In conclusion, our findings strongly indicate that survivin suppression induces mitochondrial apoptosis in human MFH/UPS cells, and that YM155 exhibits antiproliferative activity and induces tumor regression in human MFH/UPS xenografts. We propose that survivin suppressants, including YM155, be considered as potent therapeutic agents for the novel treatment of human MFH/UPS.
